# Supplementary material for: Metabolic, Affective and Neurocognitive Characterization of Metabolic Syndrome Patients with and without Food Addiction. Implications for Weight Progression
Source: Nutrients. 2021 Aug 13;13(8):2779. doi: 10.3390/nu13082779 (PMC8398101; doi:10.3390/nu13082779)
Supplement: Supplementary file 1 [file nutrients-13-02779-s001.zip › nutrients-1319424-supplementary.pdf]

Table S1 (supplementary material). Evolution of BMI (kg/m^2) during the study: Mixed 2×3 ANOVA

	**Descriptives**							**Multivariate Tests**			
	**Baseline (BS)**		**1-yr (1yr)**		**3-yrs (3yr)**			***F***	***df***	***p***	η^2
Group	*Mean*	*SD*	*Mean*	*SD*	*Mean*	*SD*	Time-by-Group	4.18	2;431	**0.021***	0.010
FA –	32.35	3.38	30.83	3.89	31.25	3.73	Time	7.94	2;431	**0.001***	0.018
FA +	35.07	3.14	32.38	7.61	34.11	2.97	Group	11.43	1;431	**0.001***	0.026
Contrast	Time (within FA–)			*MD*	*p*	η^2	Time (within FA+)	*MD*	*p*	η^2	
	BS vs 1yr			1.52	**0.001***	.328	BS vs 1yr	2.69	**0.001***	.058	
	1yr vs 3yr			-0.42	**0.002***		1yr vs 3yr	-1.73	**0.001***		
	Polynomial tests			*p*	η^2		Polynomial tests	*p*	η^2		
	Lineal trend			0.062	.009		Lineal trend	0.321	0.043		
	Quadratic trend			**0.004***	.020		Quadratic trend	0.850	0.002		
Contrast	Group			*MD*	*p*	η^2					
	FA+ vs FA– (BS)			2.72	**0.001***	.034					
	FA+ vs FA– (1yr)			1.55	0.064	.008					
	FA+ vs FA– (3yr)			2.86	**0.001***	.033					

Note. FA–: food addiction negative screening. FA+: food addiction positive screening. SD: standard deviation. df: degrees of freedom. η^2: partial eta- squared. *Bold: significant comparison. Sample size: n = 434。
